# Biochemical and biological validations of a faecal glucocorticoid metabolite assay in mandrills (*Mandrillus sphinx*)

**DOI:** 10.1093/conphys/coz032

**Published:** 2019-09-05

**Authors:** Shana R Lavin, Miles C Woodruff, Rebeca Atencia, Debby Cox, Glenn T Woodruff, Joanna M Setchell, Catharine J Wheaton

**Affiliations:** 1 Animals, Science and Environment, Disney’s Animal Kingdom®, Lake Buena Vista, FL, USA; 2 Anthropology Department and Behavior, Ecology and Evolution Research Centre, Durham University, Durham, UK; 3 The Jane Goodall Institute, Vienna, VA, USA

**Keywords:** Enzyme immunoassay, HPLC, reintroduction, steroid, stress, welfare

## Abstract

Stress is a major factor in determining success when releasing endangered species into the wild but is often overlooked. Mandrills (*Mandrills sphinx*) are vulnerable to extinction due to habitat loss and demand for bush meat and the pet trade. To help bolster *in situ* populations, rehabilitated rescued mandrills recently were released into a protected area in the Republic of Congo. The goal of this study was to validate the use of faecal glucocorticoid metabolite enzyme immunoassays (EIAs) in mandrills and test field-friendly faecal hormone extraction techniques that can subsequently be used to monitor the stress physiology and welfare of mandrills throughout the release process. Using faecal samples collected from *ex situ* mandrills, we tested cortisol, corticosterone, 11β-hydroxyetiocholanolone (69a), and 11-oxoetiocholanolone EIAs. Absolute concentrations, hormone profiles following medical procedures or translocation, and high-performance liquid chromatography fraction immunoreactivity showed that the 69a assay was the best choice to monitor the stress response in this species. Samples with delayed extraction or drying times had 40–80% lower 69a concentrations than samples extracted immediately post-collection and frozen. The 69a EIA is an appropriate assay for monitoring welfare in this species *in situ* or *ex situ*, and results indicated that consistent extraction methods are important for accurate comparisons.

## Teaser

Stress is a major factor when releasing endangered species into the wild but is often overlooked. We explored methods to measure stress non-invasively that can be applied to rehabilitated rescued mandrills following release into the wild. We validated an assay for this purpose and showed that consistent extraction methods are needed for accurate hormone comparisons in this species.

## Introduction

There is an inevitable and direct link between animal translocation and physiological indicators of stress (reviewed in Dickens *et al.*, 2010). Although stress is a major factor in determining success when releasing endangered species into the wild, it is often overlooked (Teixeira *et al.*, 2007). With suitable validations and careful consideration of methodological caveats (Millspaugh and Washburn, 2004; Shutt *et al.*, 2012; Behringer and Deschner, 2017), non-invasive endocrinological techniques such as monitoring faecal glucocorticoid metabolite (FGM) concentrations can be used to assess responses to the social and environmental stressors associated with translocation and release. For example, studies of various species report increases in FGMs after translocations: eastern grey squirrel (*Sciurus carolinensis*: Bosson *et al.*, 2013), eastern bettong (*Bettongia gaimardi*: Batson *et al.*, 2017), European wild rabbits (*Oryctolagus cuniculus*: Cabezas *et al.*, 2007), Grevy’s zebra (*Equus grevyi*: Franceschini *et al.*, 2008), Przewalski’s horse (*Equus ferus przewalskii*: Ji *et al.*, 2013), greater rhea (*Rhea americana*: Lèche *et al.*, 2016), northern river otter (*Lontra canadensis*: Taylor *et al.*, 2016), and African elephant (*Loxodonta africana*: Viljoen *et al.*, 2008). Additionally, there is a direct relationship between FGMs and mortality in some species (e.g. squirrels). Such studies can identify acclimation times or a lack of acclimation (e.g. rheas), highlight critical periods when stress biomarkers are highest during the translocation process and guide future relocation efforts.

Approximately 60% of primate species are threatened with extinction, and ~75% have declining populations (Estrada *et al.*, 2017). Release projects are common because confiscated animals accumulate in sanctuaries. Only one study, however, has examined FGMs in non-human primates during releases into the wild (mantled howler monkeys; *Alouatta palliata mexicana*) and found that faecal corticosterone metabolite concentrations were lower than prior to translocation (Aguilar-Cucurachi *et al.*, 2010). The authors indicated that the new environment could be less stressful to these monkeys than the disturbed pre-translocation habitat.

Mandrills (*Mandrills sphinx*) are found in Cameroon, Gabon, Equatorial Guinea and the Republic of Congo (Grubb, 1973). In addition to habitat loss, mandrills are hunted heavily for bush meat, and infants are vulnerable to capture for the pet trade (Sabater-Pí, 1972; Harrison, 1988; Nebasifu, 2015); the International Union for Conservation of Nature (IUCN) lists mandrills as vulnerable (Oates and Butynski, 2008). The Jane Goodall Institute’s Tchimpounga Chimpanzee Rehabilitation Center (Pointe Noire, Republic of Congo) has rehabilitated orphaned wild-born mandrills confiscated by, or with the approval of, the Congolese environmental law enforcement agency, the Ministère de l’Economie Forestière. In 2013–15, a subset (*n* = 14) of these mandrills was released into the Republic of Congo (Lavin *et al.*, 2015; the Jane Goodall Institute, 2016). The goal of our study was to use mandrills housed at a zoo to validate an FGM assay and field-friendly faecal hormone extraction techniques that could subsequently be applied to monitor the mandrills in Africa throughout the release process.

Research investigating stress physiology in mandrills has employed faecal cortisol assays (Setchell *et al.*, 2008, 2010; Charpentier *et al.*, 2018), but no comprehensive set of validations (Millspaugh and Washburn, 2004; Shutt *et al.*, 2012; Behringer and Deschner, 2017) for quantifying FGMs in mandrills has been published. Alternative FGM assays to those specific to native cortisol or corticosterone (e.g. 11ß-hydroxyaetiocholanolone: Frigerio *et al.*, 2004; 11-oxoaetiocholanolone: Mostl *et al.*, 2002) are more sensitive in detecting physiological responses following stressful situations in many species including primates (e.g. Wasser *et al.*, 2000; Heistermann *et al.*, 2006; Fichtel *et al.*, 2007; Pirovino *et al.*, 2011; Weingrill *et al.*, 2011; Shutt *et al.*, 2012; however, see Wheeler *et al.*, 2013), and this may also be the case in mandrills. Validating and employing an appropriate faecal hormone extraction and quantitative method of monitoring stress and ensuring acclimation in individual mandrills during repatriation events would strengthen the science underlying the reintroduction biology of this species (Seddon *et al.*, 2007; Armstrong and Seddon, 2008) and guide subsequent repatriation monitoring protocols. In our study, we validated the use of an FGM enzyme immunoassay (EIA) in zoo-housed mandrills and tested field-friendly faecal hormone extraction techniques to monitor animal welfare. We hypothesized that group-specific assays would be more sensitive than cortisol or corticosterone assays, and varying extraction methods would affect hormone metabolite concentrations.

## Materials and methods

### Sample collection

We collected faecal samples opportunistically from three mandrills (1♂ and 2♀) at Disney’s Animal Kingdom^®^ with housing conditions described previously (Phillips and Wheaton, 2008) coinciding with veterinary procedures (female #1, anaesthetized for diagnostic examination due to menstrual discomfort; female #2, anaesthetized for routine examination) or institutional transfer (male) as veterinary immobilizations and translocations between zoos elicit adrenal responses in primates (e.g. Heistermann *et al.*, 2006; Wark *et al.*, 2016). We collected samples from known individuals immediately after defecation on the morning of the day of the event and for 5–7 days following the event. We stored samples immediately at −20°C prior to processing. Animal diets were consistent throughout the study and consisted of low-starch primate biscuits and a variety of low-sugar produce items.

We extracted faecal samples using methods described previously (Wheaton *et al.*, 2007). Briefly, 1 ml of 80% methanol was added per 0.1 g of faeces and placed on a shaker on the low setting (Eberbach Co., Ann Arbor, MI) overnight. We centrifuged samples at 2500 rpm for 30 min at 5°C and removed and stored the supernatant at −80°C until analysis.

### EIAs

We used EIAs to screen mandrill faecal cortisol, corticosterone, and cortisol metabolites with 3α,11ß-hydroxy (Ganswindt *et al.*, 2003; Frigerio *et al.*, 2004; Heistermann *et al.*, 2006; 11β-hydroxyetiocholanolone; hereafter, `69a’) and 3α,11oxo structures (Mostl *et al.*, 2002; 11-oxoetiocholanolone; hereafter, `72t’) as putative stress biomarkers in mandrills. We also used testosterone (T5) EIAs for high-performance liquid chromatography (HPLC) validations (Section 2.2.1). We ran assays on microtiter plates (Corning 9018 CoStar 96-well EIA plates; Corning, NY) using a double antibody system.

For cortisol, corticosterone, and T5 assays, we coated plates with 150 μl goat anti-rabbit IgG (0.010 mg/ml; Arbor Assays, Ann Arbor, MI) dissolved in coating buffer (10 mM phosphate, 0.005% Proclin 150) and incubated them overnight at room temperature (RT; 22°C, 40% humidity). We emptied the wells, filled them with 250 μl blocking buffer (10 mM phosphate, 0.1% Tween 20, 0.09% sodium azide, 15 mM NaCl, 1% sucrose), and incubated them overnight at RT.

For 69a and 72t EIAs, we coated plates with 250 μl Protein A (0.002 mg/ml; Sigma-Aldrich P-7837/P-3838; St. Louis, MO) dissolved in coating buffer and incubated them overnight at RT. We emptied the wells, filled them with 300 μl blocking buffer, and incubated them overnight at RT. We emptied plates and dried them overnight at RT in a dry keeper (Sanplatec, Osaka City, Japan), packaged them with desiccant in a heat-sealed pouch (Pactech, Rochester, NY), and stored them at 4°C for use within 1 year of coating.

For cortisol, T5, and corticosterone assays, we added 50 μl standards, controls or samples to each well followed by 50 μl of horseradish peroxidase (HRP) conjugate and 50 μl antibody. We used methods described in Freeman *et al.* (2018) for cortisol (R4866) and T5 (R156/7) assays. The corticosterone assay consisted of corticosterone-HRP conjugate (1:300 000) and corticosterone antibody CJM006 (1:400 000). We placed plates on a shaker for 5 min, sealed them, incubated them overnight, and washed them 3 times with wash buffer (10 mM phosphate buffer, 0.05% Tween 20, 0.0045% Kathon, 15 mM NaCl, 1 mM EDTA). After the wash step, we added 100 μl of high kinetic tetramethylbenzidine (TMB-HK; 2.5 mmol/l; Moss Inc., Pasadena, MD) to each well and incubated plates at RT for 30 min, after which we added 50 μl of stop solution (35–38% HCl; Thermo Fisher Scientific A481–212; Pittsburgh, PA) to each well. We incubated plates at RT on a shaker for 1 min and quantified optical density using an Emax or Emax Plus plate reader (Molecular Devices LLC, Sunnyvale, CA) with a test filter of 450 nm and a reference filter of 650 nm in conjunction with SoftMax Pro software (version 6.2.2/6.4.2; Molecular Devices LLC, Sunnyvale, CA).

The 69a assay is described in Frigerio *et al.* (2004), and cross-reactivities are characterized in Ganswindt *et al.* (2003). In duplicate, we added 50 μl of standards, controls, or samples to each well followed by 100 μl of label 5ß-androstane-3α,11b-di-ol-17-one-CMO-biotinyl-LC (provided by R. Palme, Vienna Austria; diluted 1:900 000 in assay buffer) and 100 μl of antibody 5ß-androstane-3α,11b-di-ol-17-one-CMO:BSA (provided by R. Palme, Vienna Austria; diluted 1:17 000 in assay buffer).

The 72t assay is described in Mostl *et al.* (2002), and cross-reactivities are characterized in Ganswindt *et al.* (2003). In duplicate, we added 50 μl of standards, controls, or samples to each well followed by 100 μl of label 11-oxoetiocholanolone-17-CMO-biotinyl-3,6,9-trioxaundecanediamin (provided by R. Palme, Vienna Austria; diluted 1:2 000 000 in assay buffer) and 100 μl of antibody 11-oxoetiocholanolone-17-CMO:BSA (provided by R. Palme, Vienna Austria; diluted 1:60 000 in assay buffer).

We placed 69a, and 72t plates on a shaker for 5 min, sealed them, and incubated them overnight at RT. After incubation, we washed the plates 3 times and added 150 μl of streptavidin-peroxidase conjugate (0.02 U/ml assay buffer; Roche Diagnostics Co., Indianapolis, IN) to each well. After a 45-min incubation on a shaker at RT, we washed the plates, added 250 μl of TMB-HK to each well, and incubated them at RT for 30 min, after which we added 50 μl of stop solution to each well. We incubated plates at RT on a shaker for 3 min and quantified optical density as described above.

#### 69a EIA and HPLC validations

We generated standard curves (5.12–7812.5 pg/well in duplicate) for each plate using purified 69a (Steraloids A3120-000; Newport, RI). We ran 4 controls in duplicate on each plate representing a high (700 pg/well) and low (70 pg/well) concentration made from 69a standard stock solutions and mandrill sample controls representing 2 biological matrix concentrations (790 and 1700 pg/well when diluted 1:12 in assay buffer).

We diluted mandrill faecal extracts in assay buffer and used serial dilutions (1:1–1:128) of pooled faecal extracts to validate the 69a EIA and test whether the alcohol interfered with the assay at the dilutions used. Slopes for 69a were not significantly different for serial diluted pooled mandrill samples and the standard curve (*T*_3,16_ = −0.21; *P* = 0.84). We diluted sample extracts 1:4–1:50 depending on concentration to produce bindings of 20–80%. The coefficients of variation for intra-assay duplicates were <20%, with a mean of 8%, and inter-assay controls (*n* = 21, 96-well plates) were 11.30% and 16.46% for the high and low concentrations, respectively, and 14.55% and 14.18% for the high and low concentration biological samples, respectively. Assay sensitivity was 32 pg/well (90% binding). We spiked a pooled mandrill faecal extract with 69a standards ranging 5.12–1250 pg/well to assess recovery. Exogenous 69a added to mandrill faecal extract yielded 98 ± 1.5% recovery.

We used reverse-phase HPLC to separate steroid hormones/metabolites of interest and fraction samples for subsequent EIAs as described in Freeman *et al.* (2018). Briefly, we dried down samples of 1 ml each of female and male methanolic faecal extracts, reconstituted them in 150 μl of 40% acetonitrile, and sonicated them for 15 min. We injected a volume of 100 μl reconstituted extract onto a Hypersil GOLD™ C18 Selectivity 30 mm LC column (3 μm particle size; Thermo Fisher Scientific, Waltham, MA) with an isocratic mobile phase consisting of 40% acetonitrile (HPLC grade; > 99.99%; Thermo Fisher Scientific A998; Pittsburgh, PA) and 60% water (GenPure water filtration system; Thermo Fisher Scientific, Waltham, MA) at a flow rate of 0.25 ml/min. We collected post-column fractions every 60 seconds, dried them down using compressed air at RT, and reconstituted them in 500 μl of assay buffer. We assayed reconstituted HPLC fractions immediately using 69a, cortisol, corticosterone, and T5 EIAs (see Section 2.2).

### Field method validations

**Figure 1:**
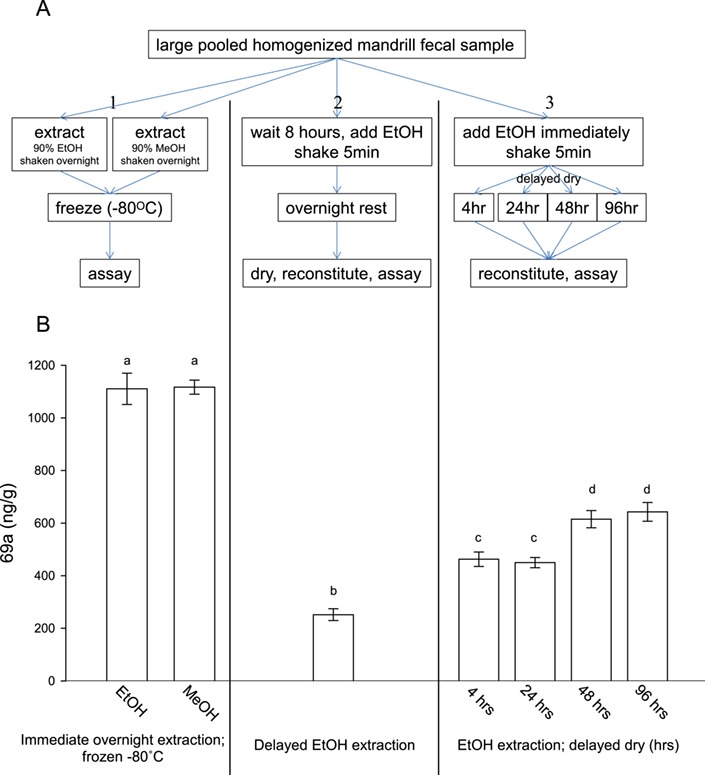
Panel **A**: experimental design to test the effects of extraction methods on faecal glucocorticoid metabolite concentrations (*n* = 10 replicates in each of 7 treatments). Experimental treatments included (i) immediate overnight extractions in 90% ethanol or 90% methanol with faecal portions removed and supernatants stored at −80°C; (ii) extractions in 90% ethanol after an 8-hour delay at RT, then removing faecal portions the following day, evaporating supernatants using compressed air at RT and reconstituting them in 80% methanol (methods practical in a field setting where faecal samples may not be collected or immediately processed); and (iii) immediate extractions in 90% ethanol with varying times before removing faecal portions and evaporating supernatants (4–96 hours after ethanol addition) using compressed air at RT followed by reconstitution in 80% methanol (the methods used in Lavin *et al.*, 2015 and the Jane Goodall Institute, 2016). Panel **B**: faecal glucocorticoid metabolite concentrations (means ± standard error of the mean) for the extraction methods in Panel A. Different letters indicate significantly different concentrations (*P* < 0.05).

We simulated field methods used to monitor mandrills *ex situ* in which samples were protected from exposure to ultraviolet light via a shade or a roof and placed immediately in ethanol and thus subject to minimal ambient humidity (Lavin *et al.*, 2015; the Jane Goodall Institute, 2016). We compared these methods with typical laboratory conditions and methods (immediate overnight extractions in 90% ethanol or 90% methanol, and frozen at −80°C, reviewed in Khan *et al.*, 2002; Pettitt *et al.*, 2007) and with a more feasible field-friendly method in which faecal samples may not be collected or processed immediately. We pooled mandrill faecal samples, homogenized them by hand, and assigned 70 subsamples of 0.5 g faeces each to 1 of 7 treatments, resulting in 10 samples per treatment. Experimental treatments included (i) immediate overnight extractions in 90% ethanol (`EtOH’) or 90% methanol (`MeOH’) with faecal portions removed and supernatants stored at −80°C (comparable to methods in Section 2.1); (ii) extractions in 90% ethanol after an 8-hour delay at RT, then removing faecal portions the following day, evaporating supernatants using compressed air at RT, and reconstituting them in 80% methanol (`delayed EtOH’; methods practical in a field setting where faecal samples may not be immediately collected or processed immediately); and (iii) immediate extraction in 90% ethanol with varying amounts of time before removing faecal portions and evaporating supernatants (4–96 hours after ethanol addition) using compressed air at RT followed by reconstitution in 80% methanol (e.g. `delayed dry 4 hours’; the methods used in Lavin *et al.*, 2015 and the Jane Goodall Institute, 2016; Fig. 1).

### Statistical analyses

We tested parallelism between serial dilutions of faecal extracts and the standard curve (*P* > 0.05 for the interaction term in the model) using a general linear model (Systat version 13; Chicago, IL). We used Shapiro–Wilk and Levene’s tests to assess normality and homogeneity of variance, respectively, and a one-way analysis of variance and *post hoc* Dunnett’s T3 tests to test for significant differences (*P* < 0.05) among sample treatments (Predictive Analytics SoftWare Statistics 18; IBM, Armonk, NY).

## Results

### Assay and HPLC validations

**Figure 2:**
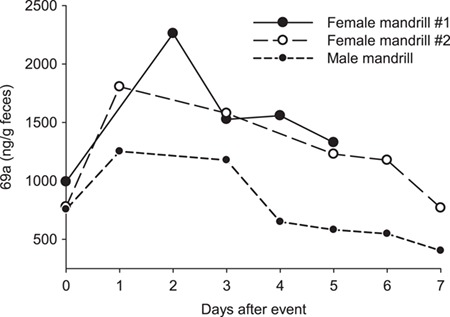
Faecal glucocorticoid metabolite profiles of male and female mandrills following a routine management stressor (institutional transfer or medical treatment) elicited on Day 0.

We detected minimal concentrations overall (<20 ng/g) and a lack of or a nominal rise in cortisol and corticosterone following management events in zoo-managed mandrills. For example, in 1 female mandrill, baseline faecal cortisol was 27.85 ng/g and baseline corticosterone was 13.37 ng/g, the latter being at the lower limit of detection for this assay. Following the routine examination with anaesthesia, faecal cortisol was 19.87 ng/g and corticosterone was 17.24 ng/g. Extracted 72t was found in relatively higher concentrations (<400 ng/g) after the procedure but not as high as 69a (>500 ng/g; Fig. 2). Thus, we did not pursue cortisol, corticosterone, and 72t assays further for use in this species; although, we incorporated cortisol and corticosterone assays in subsequent HPLC validations. Faecal 69a was 2–2.5× higher 1–2 days following management events for both male and female mandrills (Fig. 2).

**Figure 3:**
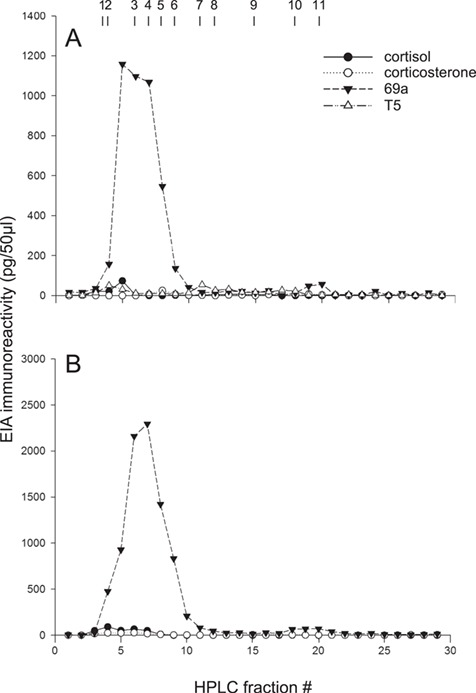
EIA immunoreactivity of fractions separated by HPLC for cortisol (filled circles), corticosterone (open circles), 69a (filled triangles) and T5 (open triangles) in faecal extracts from male (Panel **A**) and female (Panel **B**) zoo-managed mandrills. Numbered arrows indicate HPLC elution times for steroid standards: aldosterone (1), cortisol/cortisone (2), corticosterone (3), 69a (4), 72t/5α-androstane-3,11,17-trione (5), 5β-androstan-3, 11, 17-trione (6), T5 (7), androstenedione/dehydroepiandrosterone (8), epiandrosterone/5ß-androstane-3ß-ol-17-one/5ß-dihydrotestosterone (9), etiocholanolone (10) and androstanedione/androsterone (11).

The HPLC elution order of steroid standards was the same as reported previously (Freeman *et al.*, 2018). Compared to the 69a EIA, both male and female faecal extract fractions had minimal immunoreactivity in cortisol, corticosterone, and T5 EIAs (<100 pg/50 μl; Fig. 3). The highest 69a immunoreactivity (1158 pg/50 μl; fraction 5) for the male faecal extract tested was at an elution time for an unidentified metabolite that was more polar (eluted earlier) than 69a, in between the polarity of cortisol/cortisone and corticosterone, and likely a glucocorticoid metabolite (Ganswindt *et al.*, 2003). Our HPLC standards included all compounds that cross-reacted with the 69a assay except etiocholanedione (5ß-androstane-3,17-dione; < 1% cross-reactivity; Ganswindt *et al.*, 2003), which was not commercially available. Its conformational isomer (5α-androstane-3,17-dione; androstanedione), however, co-eluted with androgen metabolites (Fig. 3), so etiocholanedione is not a likely candidate for the unidentified metabolite in the male mandrill’s faecal sample. There were comparable 69a immunoreactivities (~1100 pg/50 μl) in the same male faecal extract fractions as the corticosterone elution time (fraction 6; standard 3) and the 69a elution time (fraction 7; standard 4). Peak immunoreactivity for T5 (54 pg/50 μl) in the male faecal extract aligned with the elution time for T5 (fraction 11; standard 7), and T5 immunoreactivity (50 pg/50 μl) was comparable in the fraction where cortisol and cortisone eluted (fraction 4; standard 2). Peak cortisol (74 pg/50 μl; fraction 5) and corticosterone (25 pg/50 μl; fraction 8) immunoreactivity in male faecal extract fractions were in later fractions compared to when cortisol/cortisone (fraction 4; standard 2) and corticosterone (fraction 6; standard 3) eluted, respectively (Fig. 3A).

The female faecal extract had highest 69a assay immunoreactivity (2294 pg/50 μl) at the elution time of 69a (fraction 7; standard 4). Peak cortisol (91 pg/50 μl) and corticosterone (28 pg/50 μl) immunoreactivities were in female faecal extract fractions when cortisol/cortisone (fraction 4; standard 2) and corticosterone (fraction 6; standard 3) eluted, respectively (Fig. 3B). A fractioned sample extract from another female resulted in comparable EIA profiles (data not shown).

### Field method validation

There was a significant effect of sample treatment on 69a concentration among extraction methods (*F*_6,61_ = 95.13; *P* < 0.001; mean CV = 6.7%; Fig. 1B). Samples extracted using typical laboratory conditions and methods (immediate overnight extractions and frozen at −80°C) resulted in significantly higher 69a concentrations than samples tested with field methods that employed delayed ethanol extraction (Dunnett’s T3 test, *P* < 0.05) or ethanol extraction with delayed drying times (*P* < 0.05). The lowest 69a extraction yield occurred when samples rested at RT for 8 hours before the addition of ethanol for extraction (*P* < 0.05). Samples that stayed in ethanol for longer (>48 hours) had significantly higher 69a concentrations than those held for shorter intervals (<24 hours; *P* < 0.05; Fig. 1B).

## Discussion

Given the vulnerable status of mandrills (Oates and Butynski, 2008), maintaining *ex situ* assurance populations in zoos is important, as are carefully planned release efforts. Monitoring FGM concentrations in individual animals is a valuable tool to monitor stress physiology and ensure positive welfare during translocation and after release which may increase the chance of reintroduction success and is consistent with IUCN guidelines following animal translocation or reintroduction (Teixeira *et al.*, 2007; International Union for Conservation of Nature/SSC, 2013).

In this study, we sought to identify and validate a suitable EIA assay to monitor welfare in mandrills following release in the Republic of Congo. To our knowledge, three studies exist investigating FGM concentrations in this species (Setchell *et al.*, 2008, 2010; Charpentier *et al.*, 2018). In one of these studies (Setchell *et al.*, 2008), faecal cortisol concentrations were ~ 1.8 ng/mg dry mass in cycling females and increased ~ 10% during pregnancy. In male mandrills, Setchell *et al.* (2010) reported cortisol concentrations of ~ 0.07 ng/mg dry mass, which increased ~ 12% in unstable dominance hierarchies or with receptive females present. Using the same cortisol antiserum, we found that a cycling female had cortisol concentrations ~ 0.02 ng/mg wet mass, which did not increase following a stressful event. Differences in cortisol values could be attributed to differences in extraction or assay methods and mathematical corrections employed (Setchell *et al.*, 2008, 2010).

Based on mounting evidence of increased abundance and improved sensitivity using group-specific assays that measure hormone metabolites rather than native cortisol in primate faecal samples (e.g. Wasser *et al.*, 2000; Heistermann *et al.*, 2006; Fichtel *et al.*, 2007; Pirovino *et al.*, 2011; Weingrill *et al.*, 2011; Shutt *et al.*, 2012; however, see Wheeler *et al.*, 2013), we pursued alternative assays to cortisol in mandrill faecal samples. Specifically, we also tested corticosterone, 72t, and 69a; the last of which proved to be more abundant and sensitive in our tests. Both analytical (parallelism and spike recovery) and biological (increased concentrations following an event predicted to elicit a stress response) validations supported the use of the 69a assay. This assay may also be more appropriate than native cortisol assays for additional primate species not yet tested and would need to be validated accordingly (e.g. this study; Shutt *et al.*, 2012).

The male mandrill had a smaller rise in 69a concentrations compared to the two females tested possibly because translocation was less stressful for the male than were the medical procedures for the females; however, all three animals had at least almost double FGM concentrations following a stressful event. These increases are more dramatic than those reported previously in association with social group dynamics or reproductive status in this species (Setchell *et al.*, 2008, 2010). Subsequent EIAs on samples fractioned by HPLC showed that peak 69a immunoreactivities were from fractions eluting simultaneously or nearly simultaneously with glucocorticoid standards and did not cross-react with androgens. HPLC fractions eluting with glucocorticoid metabolites cross-reacted with the T5 assay to the same extent as the fraction eluting with T5 but represented only 13% of the total immunoreactivity. This finding is consistent with caveats for measuring hormones non-invasively (reviewed in Goymann, 2012) and should be considered when quantifying T5 using this assay in mandrills and other species.

Sample handling protocols and extraction techniques can greatly affect concentrations and must be tested against the `gold standard’ of immediately freezing and subsequent extraction in alcohol to minimize variability (Khan *et al.*, 2002; Pettitt *et al.*, 2007). Accordingly, we tested multiple faecal extraction methods, including the field methods used for mandrills released in the Republic of Congo on FGM concentrations in mandrills (e.g. `delayed dry 4 hours’; Lavin *et al.*, 2015; the Jane Goodall Institute, 2016). Delayed faecal sample drying or delayed extraction, which could be considered typical in a field setting, consistently reduced 69a concentrations in mandrill faecal extracts by 40–80%, which could be attributed to bacterial metabolism of steroids in samples that were not frozen immediately (reviewed in Millspaugh and Washburn, 2004). Delayed faecal collection was associated with reduced FGMs in gorillas (Shutt *et al.*, 2012), but the opposite has been reported in other species due to differential cross-reactivity of the antibody to those metabolites (e.g. Mostl *et al.*, 2002). Furthermore, longer extraction times may account for the greater concentrations in the samples extracted overnight. Thus, consistent field methods, including time to extraction and extraction times, are important for accurate hormone comparisons in mandrills. It would also be helpful to determine a practical minimum and maximum extraction window of time that would yield consistent concentrations. If possible in the field, immediately adding an alcohol and extracting overnight would yield higher concentrations of glucocorticoid metabolites. Additional modifications to the extraction protocol (e.g. drying samples at higher temperatures and exposing samples to ultraviolet light and/or humidity) and variations in diets within or among individuals may also affect hormone concentrations.

In conclusion, measuring FGM concentrations using the 69a assay is an effective tool for monitoring the stress response of mandrills *ex situ* or during translocation and release into the wild. Consistent methods of sample collection and extraction are needed for accurate comparisons over time within and among individuals.
